# Itaconate and its derivatives as anti-pathogenic agents

**DOI:** 10.1039/d4ra08298b

**Published:** 2025-02-10

**Authors:** Rizkallah Al Akiki Dit Al Mazraani, Naglis Malys, Vida Maliene

**Affiliations:** a Bioprocess Research Centre, Faculty of Chemical Technology, Kaunas University of Technology Radvilėnų st. 19 Kaunas LT-50254 Lithuania; b Department of Organic Chemistry, Faculty of Chemical Technology, Kaunas University of Technology Radvilėnų st. 19 Kaunas LT-50254 Lithuania n.malys@gmail.com; c Built Environment and Sustainable Technologies Research Institute, Faculty of Health, Innovation, Technology and Science, Liverpool John Moores University Byrom Street Liverpool L3 3AF UK

## Abstract

Pathogenic microorganisms and viruses cause outbreaks and pandemics that affect millions of people worldwide. Despite recent advances in pharmacology and medicine, the ability of infectious diseases to spread in the modern era is accelerating due to various factors contributing to increased human-to-human and human–animal contacts. With the global rise of drug resistance among pathogens and frequently occurring viral outbreaks, alternative drugs and therapies that specifically inhibit microbial virulence or regulate immune responses are attracting growing interest. The present review focuses on itaconate and its derivatives as potential anti-pathogenic agents. It summarizes the current state of research on itaconate metabolism in bacteria, fungi and mammals. This is followed by a comprehensive review of recent advances studying itaconate and its derivatives as anti-inflammatory, immunoregulatory, antimicrobial and antiviral compounds, along with their mechanisms of action. Finally, the review emphasises the existing challenges and future research directions for the application of itaconate and its derivatives as anti-pathogenic agents.

## Abstract 2

Itaconate and its derivatives.

## Graphical abstract

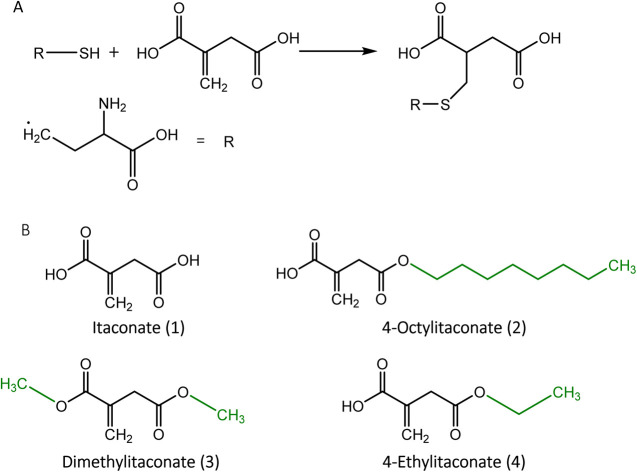

## Introduction

Itaconate is a versatile compound with a wide range of applications, including its use as a monomer in the synthesis of plastics and food packaging.^1–3^ Importantly, it exhibits anti-pathogenic^4,5^ and immunoregulatory properties.^6^ Itaconate was originally discovered by Gustav Crasso and Jean Louis Laissaigne around 1840,^7^ who revealed that itaconate is formed from aconitate while studying the thermal decomposition of citric acid. The itaconate biosynthesis by the fungus *Aspergillus* was first reported nearly a hundred years later in 1931.^8–10^ Furthermore, the biosynthesis of itaconate at an industrial scale has been demonstrated through the whole-cell bioconversion of citrate to itaconate in engineered *Escherichia coli*.^11,12^ The chemical synthesis of itaconate by the carbonization of citric acid, followed by hydrolyzation of the anhydride, has been attempted to increase the yield for industrial needs.^13^ However, no chemical method can rival fungal production of itaconate.^14^

Over a decade ago, mammalian immune cells, specifically macrophages, were shown to produce itaconate in mitochondria in large quantities in response to activation by the inflammatory stimulus lipopolysaccharide (LPS).^15–17^ The *cis*-aconitate decarboxylase (ACOD1), encoded by the immunoresponsive gene 1 (*Irg1*), was identified as the enzyme responsible for catalysing the decarboxylation of *cis*-aconitate, a tricarboxylic acid (TCA) cycle intermediate, to itaconate.^18^ Since its emergence as a mitochondrial metabolite with inflammation-regulatory properties, interest in itaconate has grown significantly.^19–23^

With the increase in pathogen resistance to conventional medical treatments and the recent severe acute respiratory syndrome coronavirus 2 (SARS-CoV-2) pandemic,^24–28^ the immunomodulatory, antimicrobial and anti-pathogenic properties of itaconate have attracted substantial research.^29–33^ Itaconate and its derivatives have also been demonstrated to possess antiviral properties.^22^

This review will delve into the anti-pathogenic properties of itaconate and its derivatives. Their inhibitory effects will be discussed in detail, although not all mechanisms of action are fully understood. The medicinal potential of itaconate and its derivatives as anti-inflammatory, antimicrobial and antiviral agents will be explored. Additionally, we will examine recent discoveries that may shed light on the mode of action of itaconate derivatives as antiviral agents, potentially explaining previously observed effects where the mechanisms were unknown.

### Itaconate's chemical properties in the biological context

**Fig. 1:**
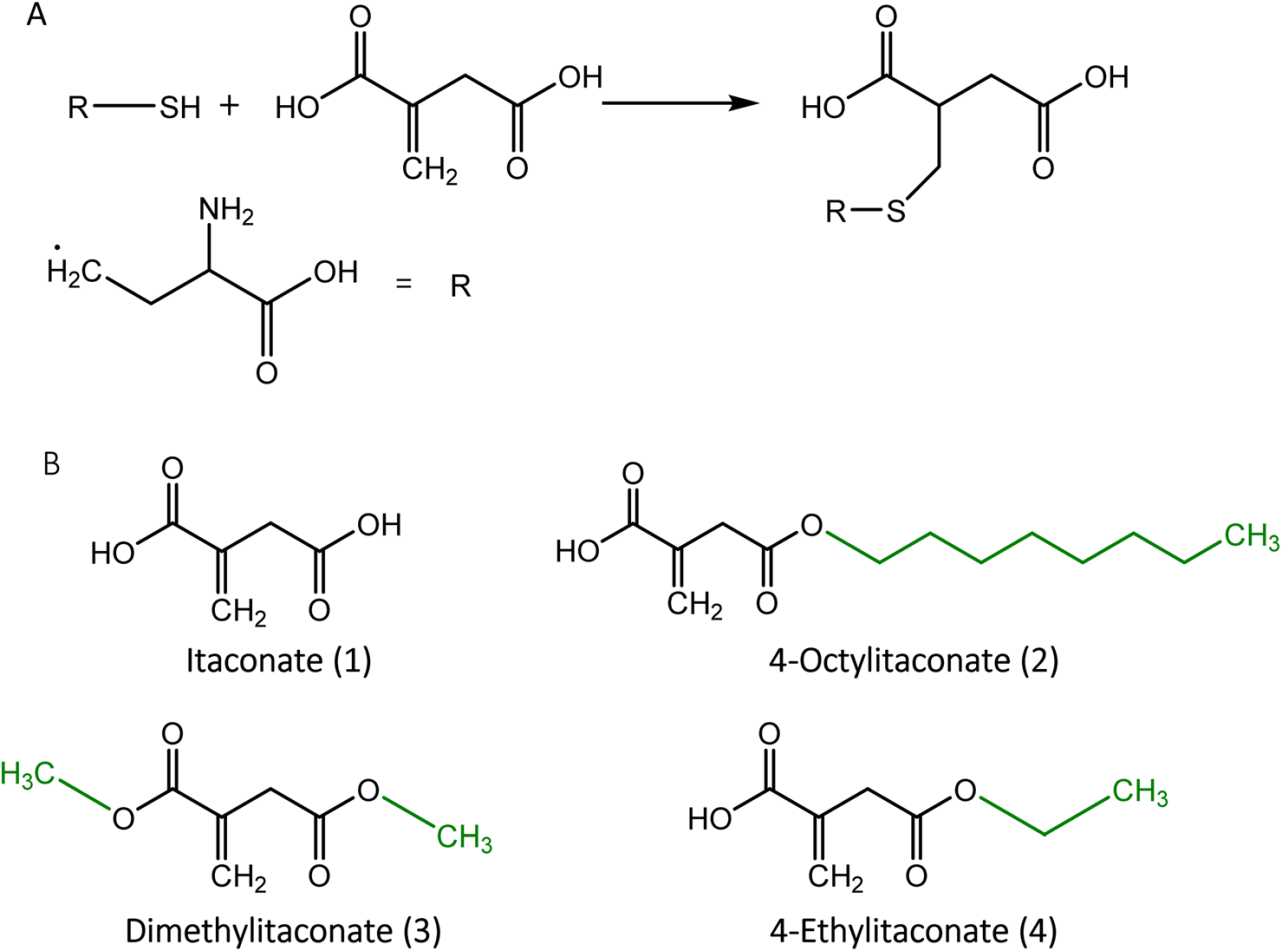
(A) Alkylation of cysteine by itaconate. (B) Structural representation of itaconate and its derivatives. Residues used to generate itaconate derivatives are shown in green.

Itaconate (1) is an α,β-unsaturated dicarboxylic acid (C_5_H_6_O_4_) with a characteristic double bond and two carboxyl groups. The unsaturated double bond can accept an electron pair, enabling it to act as an acceptor in the Michael reaction.^34^ Due to this chemical property, itaconate can alkylate the cysteine residue (Fig. 1) of the peptide.

Similar to other dicarboxylic acids, itaconate exhibits strong polarity and hydrophilicity, causing weak permeability across the membrane,^35^ which hinders the investigation of its effects in the cell. To improve itaconate's ability to enter the cell, esterification was applied to derive substitute compounds such as 4-octylitaconate (2) (4-OI),^35^ dimethylitaconate (3) (DI)^19^ and 4-ethylitaconate (4) (4-EI).^36,37^ The most studied derivative, 4-OI, was found to be converted to itaconate upon entry into the cell by the esterase,^36^ whereas DI exhibited a short-term effect without the formation of itaconate and was metabolized rapidly in the cell.^38^ DI also showed a wide spectrum of effects on metabolism caused by the covalent modification of metabolic enzymes.^37^ Although 4-EI has a similar structure and polarity to DI, its effect was less pronounced in the cell.^39^

### Chemical synthesis of itaconate and its derivatives

**Fig. 2:**
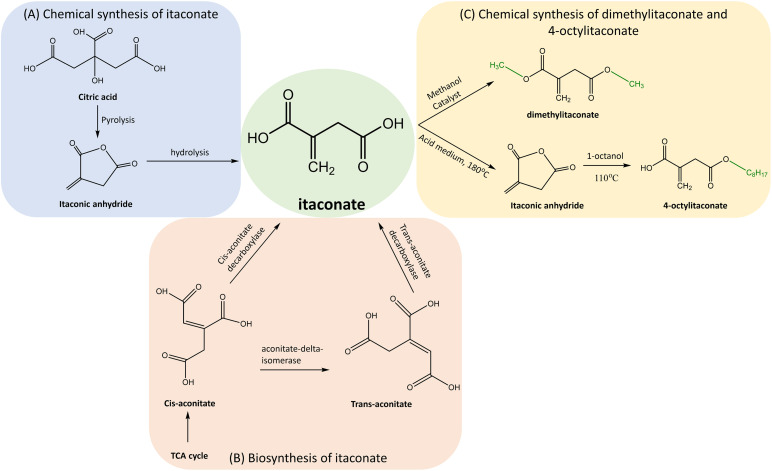
Synthesis of itaconate and its derivatives. (A) Chemical synthesis of itaconate by pyrolysis of citric acid followed by hydrolysis of itaconic anhydride. (B) Biosynthesis of itaconate in fungi from *cis*-aconitate by *cis*-aconitate decarboxylase, either directly or by passing through *trans*-aconitate. (C) Chemical synthesis of itaconate derivatives DI and 4-OI by esterification of itaconate.

Various chemical methods were developed for the synthesis of itaconate. The pyrolysis of citric acid followed by hydrolysis of itaconic anhydride (Fig. 2) was one of the early methods developed by Baup.^40^ Later, the carboxylation of aconitic acid was introduced by Crasso.^7^ Other variations of chemical synthesis of itaconate were developed. However, neither of these chemical synthesis methods cannot rival the microbial biosynthesis using fungi, which is currently preferred for the commercial production of itaconate.

The itaconate derivatives such as 4OI and DI can be chemically synthesised by esterification of itaconate. The early synthesis of 4-OI described by Gargallo *et al.*^41^ was based on one-step esterification by 1-octanol and using an acid as a catalyst. This method was not environment-friendly and presented a low yield of 35%. Therefore, a two-step method was developed.^42^ It involved convertion of itaconate to itaconic anhydride using sulphuric acid (H_2_SO_4_) as a catalyst at a temperature of 180 °C and pressure of 10 mm Hg followed by anhydride's esterification to 4-OI by 1-octanol at a temperature of 110 °C. Although the yield increased to 95% and selectivity for the derivative was 94%, the itaconic anhydride was susceptible to the hydrolysis and the production cost was high. In order to reduce the cost and increase the selectivity, a novel method was developed.^43^ The esterification by 1-octanol was applied in the presence of toluene and immobilized lipase (Novozyme 435) at a temperature of 50 °C achieving the 4-OI's yield of 99% with a 99% selectivity.

DI can also be synthesized by the esterification of itaconate with methanol and using H_2_SO_4_ as the catalyst.^44^ Other catalysts such as La^3+^ ∼ SO_4_^2−^/TiO_2_–SiO_2_ and Ce^4+^ ∼ SO_4_^2−^/TiO_2_–SiO_2_ were applied to achieve the yield of 94.31% and 93.43%, respectively.

### Metabolism of itaconate

#### Synthesis of itaconate in mammalian cells

**Fig. 3:**
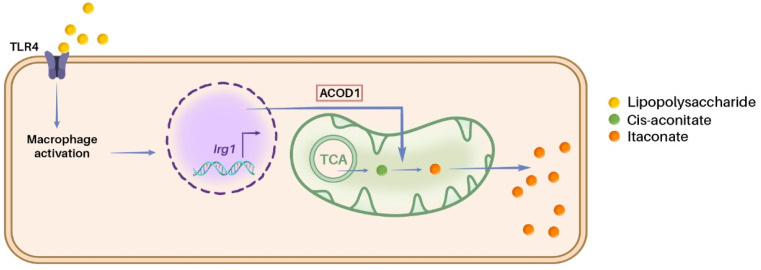
Biosynthesis of itaconate in macrophages. The TLR4 receptor, activated by LPS from Gram-negative bacteria, triggers the upregulation of *Irg1* expression, encoding ACOD1, which leads to itaconate biosynthesis in macrophages.

In macrophages, the production of itaconate can be activated by the classical Toll-like receptor 4 (TLR4)-ligand LPS (Fig. 3). It induces the upregulation of *Irg1* gene expression in a Toll-IL-1 receptor domain-containing adaptor inducing IFN-β (TRIF)/interferon regulatory factor 1 (IRF1)-dependent manner, leading to the synthesis of ACOD1.^45,46^ Subsequently, this enzyme catalyzes the production of itaconate from the TCA cycle intermediate *cis*-aconitate^18^ Similarly, *Irg1* expression-induced production of itaconate was also reported in neutrophils and neuronal cells.^5,47^ Interestingly, citraconate, a naturally occurring isomer of itaconate, was identified as an ACOD1 inhibitor.^48^

#### Synthesis of itaconate in fungi

Amongst microorganisms, the natural production of itaconate is widespread in fungi, with *Aspergillus terreus* being the most studied.^49^ This ascomycete garnered attention for industrial itaconic acid production due to its high tolerance to low pH and impressive product yields.^50^ Additionally, *Ustilago maydis* proved valuable for itaconic acid production,^51^ leveraging its yeast-like morphology advantageous for large-scale manufacturing. However, addressing low yield and limited pH tolerance required metabolic engineering and strain selection.

Despite shared transport mechanisms for itaconate production in both fungi, their metabolic pathways diverge. *A. terreus* features *cis*-aconitate decarboxylase,^52^ whereas *U. maydis* expresses aconitate-delta-isomerase in conjunction with *trans*-aconitate decarboxylase for the conversion of *cis*-aconitate to itaconate^51^ (Fig. 2).

#### Catabolism of itaconate in bacteria

The catabolism of itaconate was studied in different bacteria including *Micrococcus* spp., *Salmonella* spp., *Yersinia* spp., and *Pseudomonas* spp.^53–55^ The *in vitro* itaconate-degradation pathway was reconstructed for pathogens such as *Yersinia pestis* and *Pseudomonas aeruginosa* by characterizing key enzymes, namely, itaconate coenzyme A (CoA) transferase, itaconalyl-CoA hydratase and (*S*)-citramalyl-CoA lyase, required for itaconate degradation to acetyl-CoA and pyruvate.^4^ The genes *ict*, *ich* and *ccl* encoding the corresponding enzymes were found to be essential for the survival of pathogens in macrophages. In addition, acyl-CoA dehydrogenase, a glyoxalase family protein, and an MmgE–PrpD family protein were implicated to contribute to itaconate catabolism.^4^

### Metabolic engineering for the biosynthesis of itaconate

Itaconate has been commercially produced using the filamentous fungus *A. terreus*.^56^ Despite achieving a 60% yield, the interest in improving the productivity of this process for industrial use remains high. Several microorganisms, including *A. terreus*, *Ustilago* sp. and *Candida* sp., have the capability to produce itaconic acid, but their production levels are low using glucose as a carbon source.^55,57–60^ The yield of itaconate varies depending on several factors, mainly the carbon source. When mannose was used with *A. terreus*, the yield of itaconate was 0.46 g g^−1^;^59^ however, when glucose was used as the carbon source with *A. terreus*, the yield increased to 0.62 g g^−1^.^61^ Therefore, in this section, we will discuss the metabolic engineering methods used to synthesize itaconate.

To address industrial needs, recombinant production strategies were applied for itaconate production. This involved genetic engineering and overexpression of *A. terreus* genes in *E. coli*^62^ and *Corynebacterium glutamicum*.^63^ Though the native *E. coli* lacks the *cis*-aconitate decarboxylase gene and ability to synthesize itaconate,^12^ it was engineered for this dicarboxylic acid production. In one of the studies, itaconate synthesis was achieved using glucose as a substrate and integrating the *cis*-aconitate decarboxylase gene *cad1* from *A. terreus*.^62^ In addition, genes of citrate synthase and aconitase from *C. glutamicum* were heterologously expressed and the gene encoding lactate dehydrogenase was inactivated, resulting in an itaconate yield of 0.09 mol per mol glucose.^62^ In another study, a modified strategy was explored by integrating aconitase gene from *C. glutamicum* and *cad1* from *A. terreus* and utilizing citric acid as a source.

*C. glutamicum*, which naturally tolerates itaconate well, was also engineered for the production of this compound.^63,64^ By reducing isocitrate dehydrogenase activity and integrating *cad1* gene from *A. terreus*, the yields of 0.29 and 0.02 g g^−1^ were achieved using glucose and acetate as carbon sources, respectively.

### Anti-pathogenic activities of itaconate and its derivatives

#### Inflammation-related activities of itaconate

Inflammation is one of essential biological responses of tissues and cells to harmful stimuli including pathogens. The inflammation-related activities of itaconate have come into research focus recently.^19,37,65–67^ Itaconate has been shown to inhibit pro-inflammatory interleukins, most notably, IL-1β and IL-2, by inhibiting their activators KEAP1, IκBζ, inflammasome NLRP3, GAPDH, SDH and STING. The inhibitory effect of itaconate on the glycolysis and TCA cycle of pathogens is also associated with its anti-inflammatory role. The regulatory mechanisms of itaconate in the context of inflammation are discussed below in more detail.

#### Dissociation of the KEAP1–Nrf2 complex

**Fig. 4:**
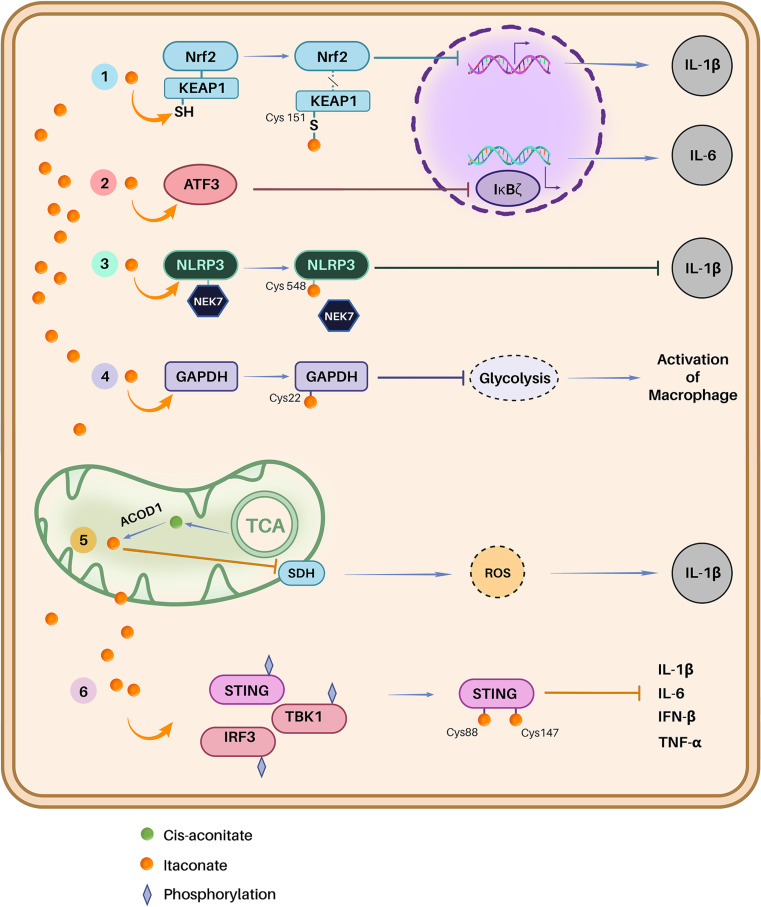
Effects of itaconate on anti-inflammatory signalling pathways. Itaconate (1) alkylates Cys151 of KEAP1, leading to the dissociation of the KEAP1–Nrf2 complex and subsequent translocation of Nrf2 to the cell nucleus, (2) increases ATF3 protein levels, facilitating its translocation to the cell nucleus, where it inhibits IκBζ at the translational level, (3) disrupts the NLRP3–NEK7 interaction through a modification known as decarboxypropylation on C548 of NLRP3, thereby preventing NLRP3-dependent IL-1β release, (4) inhibits glycolysis by alkylating Cys22 of GAPDH, (5) inhibits succinate dehydrogenase due to its structural resemblance to succinate, and (6) alkylates STING, causing a decrease in its phosphorylation and exerting an anti-inflammatory effect.

Ground-breaking research by O'Neill *et al.* has shown that itaconate can contribute to the activation of an anti-inflammatory response involving the interleukin IL-1β in the Nrf2 pathway.^36^ In this study, it has been shown that itaconate through the alkylation reaction can form the covalent bond with the cysteine residue Cys151 of Kelch-like ECH-associated protein 1 (KEAP1) (Fig. 4), leading to the dissociation of the KEAP1–Nrf2 complex and subsequent activation of Nrf2. This activation results in anti-inflammatory response, involving the inhibition of the IL-1β cleavage. Notably, although interferon (IFN-β), which promotes inflammation, triggers itaconate production, a negative feedback mechanism arises upon itaconate synthesis to inhibit IFN-β.

#### Regulation of the ATF3-IκBζ pathway

DI and 4-OI were shown to induce strong electrophilic stress that can selectively regulate secondary transcriptional response to Toll-like receptor stimulation through the inhibition of induction of a nuclear factor kappa B zeta (IκBζ) protein^37,39^ (Fig. 4), a central inflammatory regulator of the NF-KB pathway.^68^ Both itaconate derivatives inhibited IκBζ induction in an Nrf2-independent manner.^37^ DI was able to completely abolish the expression of IκBζ associated with LPS stimulation.^39^ The phosphorylation of the α-subunit of eukaryotic initiation factor 2 eIF2α, leading to protein synthesis inhibition and no change in the mRNA level of *Nfkbiz* gene encoding for IκBζ, was observed. As the eukaryotic initiation factor 2α-subunit (eIF2α) kinase plays a major role in the signaling pathway controlling IκBζ synthesis^69^ and the eIF2α phosphorylation leads to IκBζ expression inhibition; this DI effect was attributed to post transcriptional regulation. The activating transcription factor 3 (ATF3) was identified as a key mediator of IκBζ regulation.^39^

Itaconate was also shown to induce the electrophilic stress response. However, this compound showed a less immunosuppressive phenotype compared to DI and 4-OI.^37^ The comparative analysis of itaconate and its derivatives revealed divergent inflammasome and immune response in macrophages. By identifying itaconate as an immunoregulatory metabolite, this study highlights the importance of using the unmodified form of this compound in future studies.^37^

#### Effect on inflammasome's sensor NLRP3

It has been demonstrated that itaconate exerts a direct influence on NOD, LRR and pyrin domain-containing protein 3 NLRP3 (Fig. 4).^70^ NLRP3 is an inflammasome that is assembled by different proteins including the sensor NLRP3, serine/threonine-protein kinase NEK7, adaptor ASC and effector cascapase-1 (ref. 71) in order to promote inflammation by synthesizing pro-inflammatory interleukin Il-1. The inhibition occurs by the covalent bond of itaconate to Cys548.^70^ Hooftmann *et al.* study suggested that itaconate affects NLRP3 and/or NEK7 directly, or their interaction, whereas ASC or caspase-1 were not inhibited. This inhibition was proven to be a direct influence on NLRP3 by binding to Cys548. This cysteine is located in the helical domain 2 of NLRP3, which is crucial for the interaction between NLRP3 and NEK7, effectively blocking inflammasome activation and inhibiting the cleavage of IL-1β and caspase 1 into their mature forms p17 and p20, respectively. Notably, Nrf2 plays a crucial role in promoting an anti-inflammatory effect, particularly in the context of NLRP3. However, using *Nfe2l2*^*−/−*^ (a gene responsible for Nrf2 synthesis), it was observed that NLRP3 was inhibited after treatment with 4-OI.^70^

#### Inhibition of glycolysis

Itaconate and its modified form 4-OI have been revealed to inhibit aerobic glycolysis through the alkylation of glyceraldehyde-3-phosphate dehydrogenase (GAPDH) at residue Cys22(ref. 66) (Fig. 4). Inhibition of GAPDH occurred after treating the macrophage with 4-OI, which led to the inhibition of activation of the macrophage. However, to confirm that the alkylation on Cys22 is the reason of the inhibition, an overexpressed empty vector, WT GAPDH and Cys22 mutant GAPDH were used. The inhibition effect of 4-OI was observed in WT GAPDH but not in Cys22 mutant. In a similar study, a screening of the proteome with itaconate treatment was performed, and 260 cysteine residues modified by itaconate were identified.^72^ Two additional pivotal enzymes for the central metabolism were targeted by itaconate. The fructose–bisphosphate aldolase ALDOA, another enzyme implicated in the aerobic glycolysis, was alkylated in two positions of cysteine, Cys73 and Cys339, inhibiting the glucose catabolism. This study revealed that itaconate exerts an inhibitory influence on glycolysis through negative feedback regulation, leading to an anti-inflammatory effect.

#### Inhibition of TCA cycle

Itaconate was reported to inhibit the activity of succinate dehydrogenase SDH, hindering the TCA cycle to promote the anti-inflammatory effect^67,73^ (Fig. 4). It acts as a competitive inhibitor on SDH due to the similar structure of this compound to the main substrate of enzyme, a succinate.^19^ This leads to the accumulation of succinate and suppression of reactive oxygen species (ROS), resulting in the restriction of pro-inflammatory elements, including hypoxia-inducible factor-1α (HIF-1α) and diminished pro-inflammatory cytokines such as interleukin-1β (IL-1β).^74^

#### Effect on STING

Tank-binding kinase 1 (TBK1) is known to cause the phosphorylation of STING, leading to the production of interferon type I, IFN, and causing the inflammation.^75^ STING, a stimulator of interferon genes, is implicated in an inflammation storm, increasing physiological dysfunction that induces ferroptosis, promoting itself necroptosis.^76^ The endogenous production of itaconate, triggered by IFN-enhanced LPS induction of *Irg1*, inhibits STING.^77^ In a recent study, it was shown that endogenous and exogenous itaconate and its derivatives, 4-OI and DI, decrease the phosphorylation of STING, causing an anti-inflammatory effect.^78^*Irg1* expression was downregulated when transfecting with small interfering RNA for IFN regulatory factor IRF3 that is phosphorylated by TBK1, inducing the biosynthesis of IFN.^78^ Pre-treatment with the exogenous itaconate, 4-OI and DI, led to an anti-inflammatory effect, with faster conversion to itaconate derivatives due to their better membrane permeability. 4-OI was shown to inhibit the STING-based activation of IFN-β, tumour necrosis factor α TNF-α, IL-1β, and IL-6. The inhibition was due to the alkylation of STING by itaconate at several cysteine positions including Cys65, Cys71, Cys88 and Cys147. However, more detailed analysis revealed that the decrease in the phosphorylation of STING was caused by the alkylation of cysteine residues 88 and 147.

In conclusion, based on what was discussed and presented in Fig. 3, itaconate and its derivatives were able to inhibit the inflammation by binding covalently to the cysteine of proteins KEAP1, NLRP3 and GAPDH or by binding to AFT3, SDH and STING, leading to the decrease of the synthesis of interleukin IL-1β and IL-6. Interestingly, in a recent study, DI showed enhanced inflammation.^79^ This pro- and anti-inflammatory dichotomy of DI highlights the complexity of immune responses when considering derivatives of itaconate for therapeutic application.

### Antimicrobial properties of itaconate

Itaconate was proven to possess antimicrobial properties by inhibiting the bacterial growth.^80^ Itaconate inhibits key enzymes from the glyoxylate cycle such as isocitrate lyase and propionyl CoA-carboxylase, which leads to the inhibition of bacterial growth.^81,82^ In addition, itaconate was shown to activate the biosynthesis of lysosome in macrophages for the phagocytosis of pathogen.^83^ The mechanism of inhibition of enzymes as well as the activation of the biosynthesis of lysosome is explained below.

#### Inhibition of bacterial growth

**Fig. 5:**
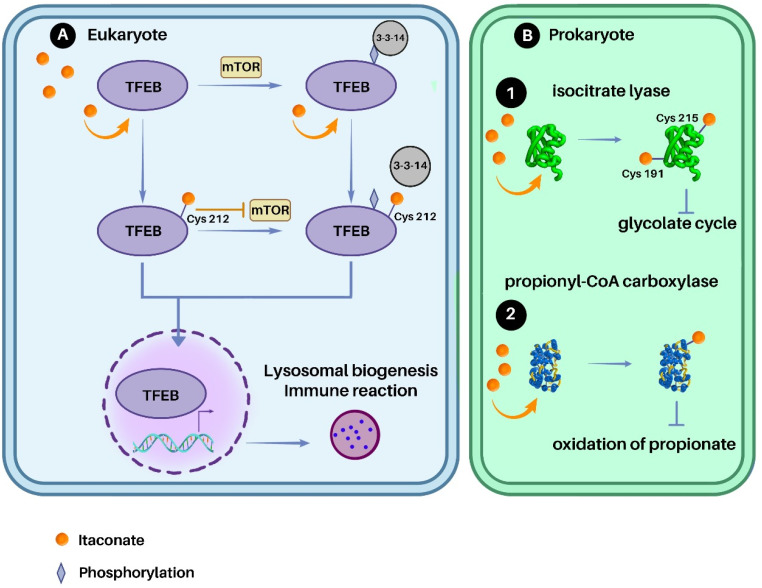
Antimicrobial effect of itaconate is shown in (A) activating macrophages and inducing the synthesis of lysosomal biogenesis by activating TFEB, and (B) inhibiting key enzymes in the glyoxylate shunt, (1) isocitrate lyase and (2) propionyl-CoA carboxylase.

Itaconate can restrict the bacterial growth by inhibiting isocitrate lyase enzyme. In *Pseudomonas indigofera*, it was shown that the kinetic activity of isocitrate lyase decreases in a dose-dependent manner with the concentration of itaconate^81^ (Fig. 5B). It wasn't until the antibacterial effect of itaconate was investigated against *Mycobacterium tuberculosis* that itaconate was shown to target cysteine residues at positions Cys191 and Cys215 within the enzyme's active site.^84^ The binding of itaconate to the enzyme was confirmed using mass spectrometry (MS/MS) analysis, following treatment with trypsin, showing that itaconate plays a possible competitive inhibitor isocitrate lyase substrate.

In addition to inhibiting isocitrate lyase, itaconate was reported to decrease the assimilation of acetate and propionate by inhibiting the activity of the propionyl-CoA carboxylase (PCAC)^82^ (Fig. 4B) in *Rhodospirillum rubrum*. Itaconate selectively inhibits the oxidation of propionate by reducing oxygen intact and competitively inhibiting PCAC since the precursor of propionate, succinate, was not inhibited. Based on the obtained data, itaconate can target other enzymes involved in the conversion of propionyl-CoA to succinate, including methylmalonyl-CoA epimerase, methylmalonyl-CoA mutase and succinate thiokinase. However, the exact mechanism of action was not fully characterized in this study.^82^

Other itaconate-related mechanisms contributing to the inhibition of pathogen growth, including those restricting the replication, have been recently studied.^32,54,85^ Studies on *Salmonella typhimurium*, *Mycobacterium avium* and other pathogenic bacteria provide links between itaconate produced in the mitochondria after stimulation of innate immune receptors and cell defence mechanisms restricting the propagation of intracellular pathogens.

#### Activation of phagocytosis

In,^86^ the itaconate was shown to play a role in the activation of the innate immune defence mechanism by facilitating the phagocytic uptake of pathogenic bacteria *via* the regulation of lysosomal biogenesis by activating Transcription Factor EB (TFEB) (Fig. 5A). TFEB was found to regulate phago-lysosome-mitochondria crosstalk in macrophages.^83^ Upon the administration of bacterial LPS from various species such as *Salmonella typhimurium*, *Escherichia coli*, and *Porphyromonas gingivalis*, the synthesis of itaconate was observed in macrophages, leading to the nuclear translocation of cytosolic TFEB protein.^86^ Immunoprecipitation coupled with liquid chromatography and mass spectrometry revealed that itaconate directly alkylates Cys212 of human TFEB. The alkylation of TFEB resulted in the inhibition of its mTOR-mediated phosphorylation at Ser211, preventing TFEB association with GST-14-3-3ζ and inducing nuclear localization. The role of itaconate as a lysosome inducer was functionally confirmed by *Irg1* knockout or expression of an alkylation-deficient TFEB, leading to the impaired antibacterial ability of macrophages.

In summary, the antimicrobial properties of itaconate extends from the direct inhibition of key bacterial metabolic pathways, targeting enzymes such as isocitrate lyase and propionyl CoA-carboxylase, to an indirect effect by triggering the synthesis of lysosomes in macrophages to activate the phagocytosis of bacteria.

### Itaconate and its derivatives as immunoregulators

The immunoregulatory role of itaconate and its derivatives has come to the attention recently with relevant research still in its pioneering phase.^37,39,87–89^ They have been recognized to suppress the inflammatory response in pro-inflammatory M1 macrophages. Furthermore, Runtsch *et al.* demonstrated that itaconate and 4-OI impede M2 macrophage polarization and metabolic remodelling by inhibiting JAK1 (Janus kinase 1) and STAT6 (signal transducer and activator of transcription 6) phosphorylation.^90^ 4-OI was shown to modify JAK1 at multiple residues including cysteines 715, 816, 943, and 1130, leading to this kinase inhibition. Using another derivative of itaconate, the DI, it was shown that a long-term trained immunity can be induced.^79^ DI was able to alter the central metabolism and mitochondrial energetics at the transcriptional, epigenomic, and metabolic levels, leading to increased responsiveness and survival to infection of pathogens such as *Staphylococcus aureus*. In addition, naturally occurring isomers mesaconate and citraconate, which have similar strong electrophile properties to itaconate, were revealed as immunoregulatory compounds.^48^

Altogether, recent pioneering studies on itaconate and its derivatives have shown their immunoregulatory potential in the treatment of immune-mediated diseases including allergy, asthma and fibrosis by targeting macrophages.

### Anti-viral properties of itaconate and its derivatives

**Fig. 6:**
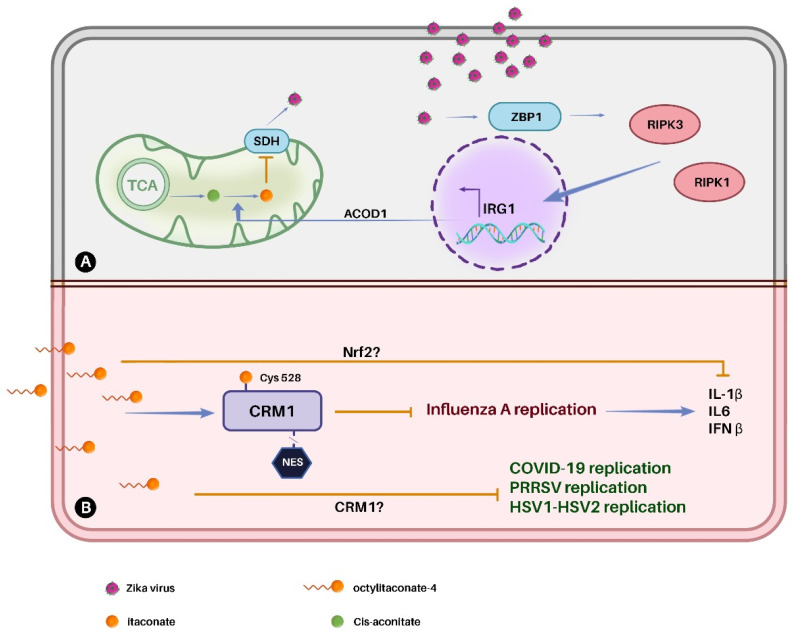
Antiviral effect of itaconate and its derivatives. (A) Neuron cells by inhibiting SDH. (B) Epithelial, kidney, and lung cells by inhibiting viral replication, which can be explained by the inhibition of CRM1 in influenza A, and may involve a similar mechanism for SARS-CoV-2, PRRSV and other viruses.

Itaconate and its derivatives have been reported to inhibit the replication of multiple viruses including influenza A, SARS-CoV-2, PRRSV (porcine reproductive and respiratory syndrome virus) and Zika.^20^ The antiviral effects of these compounds studied so far are presented in Fig. 6. Itaconate derivatives 4-OI and DI have gained the most attention as potential antiviral agents due to their cell-permeable properties. Moreover, itaconate and its naturally occurring isomers citramalate and mesaconate were shown to modulate immune response and amino acid metabolism, leading to the reduced release of viral particles of influenza A.^48^

#### Deactivation of CRM1

Chromosomal region maintenance 1 protein (CRM1), also known as exportin 1 (XPO1), is a mediator of protein nuclear export in eukaryotes.^91^ This host cell protein is exploited by viruses during their replication. 4-OI was found to inhibit the replication of influenza A virus by restricting the nuclear export of viral ribonucleoproteins through the deactivation of CRM1.^92,93^ Similar to other known CRM1 inhibitors, 4-OI modified Cys528 in the cargo binding pocket of CRM1-inhibiting protein function to bind a nuclear export signal (NES) of the cargo protein and interfering with the replication cycle of CRM1-dependent virus. In addition, it was found that itaconate and its derivatives DI and 4-OI reduced interferon responses and inflammation caused by influenza A virus infection.^94^

Considering the importance of CRM1 function to various pathogenic viruses, itaconate and its derivatives can be applied to the formulation of anti-inflammatory and antiviral therapies.

#### Inhibition of the TCA cycle

The TCA cycle plays a key role in the synthesis of ATP, amino acids and other biomolecules required for viral replication.^22^ In eukaryotes, itaconate interferes with the TCA cycle by inhibiting SDH that converts succinate into fumarate.

Itaconate has been shown to possess antiviral activity against Zika virus in neurons^47^ by inhibiting SDH (Fig. 6A). Since the neural cells have low capacity for recovery and adaptive immune response is highly regulated in the central nervous system, the effect of itaconate against Zika virus was found to be of high importance.^95^ In this study, it was observed that viral response involved the sensing of viral RNA by ZBP1 (Z-DNA-binding protein 1), which led to the activation of receptor-interacting serine/threonine kinase RIPK1 and RIPK3 regulating key inflammatory and cell death receptors.^47,96^ These activities induced *Irg1* expression, ultimately resulting in itaconate production. Knocking out genes *Zbp1* and *Ripk3* demonstrated that these genes play essential roles in the expression of the virus and inhibiting them led to virus suppression. This effect was not mediated by Nrf2, as demonstrated by qPCR analysis, which revealed no significant changes in *Nrf2* expression or its canonical targets, such as *Hmox1*, *Nqo1* and *Gclm* in *Ripk3*^*−/−*^ or *Irg1*^*−/−*^ neurons. Comparative metabolomics analysis revealed that 40 distinct metabolites exhibited significant alterations in concentration, most prominently an increase in succinate and a decrease in fumarate and malate in infected cells, which are immediate downstream products of succinate oxidation. No significant changes were observed in *Ripk3*^*−/−*^ or *Irg1*^*−/−*^ neurons. This study showed that ZBP1 and RIPK3 induced *Irg1* expression and subsequent itaconate production and TCA cycle inhibition, thus promoting antiviral metabolic state in neurons.^47^

#### Inhibition of cGAS–STING

The cGAS (cyclic guanosine monophosphate adenosine monophosphate synthase)–STING (stimulator of interferon genes) signalling pathway has emerged as a key mediator in defending against foreign pathogens and maintaining immune homeostasis.^97^ Recently, it was shown that the itaconate derivative 4-OI was capable of restricting the antiviral immune response and autoimmune inflammation by inhibiting the activation of cGAS–STING.^98^ The itaconate supplemented endogenously did not affect cGAS–STING activation, indicating divergent 4-OI and itaconate functions. At the molecular level, it was found that 4-OI directly alkylates Cys91 of STING, blocking its palmitoylation and oligomerization. Authors of this study acknowledge, however, that further research is required to understand how 4-OI alkylation or palmitoylation affects the STING activation, altering the antiviral immune response.^98^ 4-OI was used to restrict the antiviral immune response in oncolytic virotherapy using vesicular stomatitis Indiana virus VSVΔ51. 4-OI was shown to inhibit MAVS and IKKβ pathways by binding to cysteine 283 and 179 respectively,^99^ assisting in virotherapy against murine colon tumor.

#### Other cases of viral replication inhibition

Nrf2 agonist 4-OI, one of the itaconate derivative, was elucidated to induce unidentified cellular program that restricts the viral replication of SARS-CoV2 independently of type I interferons.^29^ This inhibitory effect of 4-OI extended to the replication of several other pathogenic viruses including Herpes, Vaccinia, and Zika viruses. In addition, 4-OI was implicated in a dose-dependent inhibition of PRRSV replication.^100^ Surprisingly this study showed that PRRSV can inhibit the synthesis of itaconate through the repression of the *Irg1* gene, leading to the accumulation of *cis*-aconitate.^100^

To summarise the efficacy of itaconate and its derivatives against bacteria and viruses in a dose-dependent manner, data are presented in Table 1, providing information of the minimal inhibitory concentration (MIC) of itaconate, DI and 4-OI, as well as the proposed mode of action. Both, itaconate and DI were tested against enterohemorrhagic *E. coli* and *Salmonella typhimurium*,^103^ revealing no significance in MIC (24 and 39.52 mM, respectively). Similarly, a subtle difference in MIC was observed when itaconate and DI were tested with *M. tuberculosis* (1 and 0.866 mM, respectively).^101,104^ These data suggest that there is no significant difference between the efficacies of itaconate and its derivative DI. Notably, the antimicrobial effect of DI against *M. tuberculosis*, Bacillus Calmette Guérin and multidrug-resistant *M. tuberculosis*^104^ was lower when the derivative was used directly on the bacteria than that with the macrophage.

Antipathogenic effect of itaconate and its derivatives against bacteria and viruses. Information on the MIC and the mode of action of the metabolite are provided

**Table:** 

Molecule	Bacteria	MIC (mM)	Mode of action	Reference
Itaconate	*Escherichia coli*	5	Inhibition of isocitrate lyase or proton-shuttle effect^a^	101
	*Salmonella enterica ser. Typhimurium*	20	Inhibition of isocitrate lyase or proton-shuttle effect^a^	
	*Pseudomonas aeruginosa*	20	ND	
	*Klebsiella pneumoniae*	10	ND	
	*Acinetobacter baumannii*	20	ND	
	*Enterobacter faecium*	20	ND	
	*Mycobacterium tuberculosis*	1	ND	
	*Salmonella typhimurium*	ND	ROS production	102
	Enterohemorrhagic *E. coli*	24	Inhibition of SDH	103
	*Salmonella typhimurium*	24		
DI	*Mycobacterium tuberculosis* b	0.866^b^	ND	104
	Bacillus Calmette Guérin^b^	1.2^b^	ND	
	Multidrug-resistant *Mycobacterium tuberculosis*^b^	1.2^b^	ND	
	Enterohemorrhagic *E. coli*	39.52	Inhibition of SDH	103
	*Salmonella typhimurium*	39.52		
4-OI	*Escherichia coli*	ND	ND	105
	*Salmonella typhimurium*			

a Hypothetical mode of action.

b In the article, the concentration is referred to IC_50_: half-maximal inhibitory concentration.

c The concentration when antiviral activity was observed.

**Table:** 

Molecule	Virus	MIC (mM)	Mode of action	Reference
Itaconate	ZIKA virus	ND	Inhibition of SDH	95
DI	ND	ND	ND	
4-OI	Influenza A	0.1^c^	Inhibition of CRM1	92 and 93
	COVID-19	ND	Inhibition of STAT^a^	29
	PRRSV	0.075^c^	Inhibition of STAT^a^	100

## Concluding remarks and prospects

Itaconate is an important metabolite and industrially useful chemical compound. However, the interest in its anti-pathogenic and immunoregulatory properties has come to focus recently. The comparative analysis of itaconate and its derivatives shows divergent inflammasome regulation in macrophages, revealing the immunoregulatory role of itaconate and highlighting the importance of using the unmodified version of this compound in future studies on the inflammation and immune response.^37^ On the other hand, itaconate derivatives can also find applications in therapeutic treatments. As recently shown, 4-OI can enhance oncolytic virotherapy by suppressing antiviral immunity in cancer cells through the modification of cysteine residues in the mitochondrial antiviral signalling (MAVS)-IKKβ pathway proteins.^99^ The viral replication of influenza A, a positive-strand RNA (−ssRNA) virus, occurs within the nucleus of the host. Itaconate and its derivative modify the protein CRM1 in Cys528, protein nuclear export in eukaryotes, and hindering the nuclear transportation of viral ribonucleoprotein inhibiting the replication of influenza A.^92,93^ However, SARS-CoV-2, PRRSV and Zika are positive-strand RNA (+ssRNA) viruses that replicate in the cytoplasm. Itaconate obstruct the metabolism of infected neuron cell by inhibiting SDH, demonstrating the antiviral property against ZIKA.^47^ Alternatively, NF-κB plays a crucial role in the replication of SARS-CoV-2 and PRRSV.^106–109^ NF-κB is attenuated by itaconate and its derivative either in the anti-inflammatory pathway through inhibiting the IκBζ protein^68^ or by inhibiting the inflammation caused in osteoarthrosis through the STAT-dependent NF-κB pathway.^110^ Thus, it can be hypothesized that itaconate manifests its antiviral properties against SARS-CoV-2 and PRRSV either through inhibition of NF-κB or by obstruction of cell metabolism. Future research is needed to clarify the exact mechanism. The mechanism of inhibition of viral replication by itaconate and its derivative can differ and result in contrasting antiviral and immune responses. As itaconate impacts the immune response and, particularly, type I interferons, targeting the metabolism of this compound presents new therapeutic possibilities to improve the host defense or to tackle autoimmune disorders.^88^

## Data availability

No primary research results, software or code have been included and no new data were generated or analysed as part of this review.

## Conflicts of interest

There are no conflicts to declare.
